# Exploring the sedative properties of natural molecules from hop cones (*Humulus lupulus*) as promising natural anxiolytics through GABA receptors and the human serotonin transporter

**DOI:** 10.3389/fchem.2024.1425485

**Published:** 2024-07-10

**Authors:** Amany Belal, Mohammed S. Elballal, Ahmed A. Al-Karmalawy, Ahmed H. E. Hassan, Eun Joo Roh, Mohammed M. Ghoneim, Mohamed A. M. Ali, Ahmad J. Obaidullah, Jawaher M. Alotaibi, Salwa Shaaban, Mohamed A. Elanany

**Affiliations:** ^1^ Department of Pharmaceutical Chemistry, College of Pharmacy, Taif University, Taif, Saudi Arabia; ^2^ Department of Biochemistry, Faculty of Pharmacy, Badr University in Cairo (BUC), Cairo, Egypt; ^3^ Department of Pharmaceutical Chemistry, Faculty of Pharmacy, Horus University-Egypt, New Damietta, Egypt; ^4^ Pharmaceutical Chemistry Department, Faculty of Pharmacy, Ahram Canadian University, Giza, Egypt; ^5^ Deparment of Medicinal Chemistry, Faculty of Pharmacy, Mansoura University, Mansoura, Egypt; ^6^ Chemical and Biological Integrative Research Center, Korea Institute of Science and Technology (KIST), Seoul, Republic of Korea; ^7^ Division of Bio-Medical Science and Technology, University of Science and Technology, Daejeon, Republic of Korea; ^8^ Department of Pharmacy Practice, College of Pharmacy, Almaarefa University, Ad Diriyah, Saudi Arabia; ^9^ Department of Biology, College of Science, Imam Mohammad Ibn Saud Islamic University (IMSIU), Riyadh, Saudi Arabia; ^10^ Department of Biochemistry, Faculty of Science, Ain Shams University, Cairo, Egypt; ^11^ Department of Pharmaceutical Chemistry, College of Pharmacy, King Saud University, Riyadh, Saudi Arabia; ^12^ Department of Microbiology and Immunology, Faculty of Pharmacy, Suef University, Beni-Suef, Egypt; ^13^ Department of Clinical Laboratory Sciences, Faculty of Applied Medical Sciences, King Khalid University, Abha, Saudi Arabia; ^14^ Department of Pharmaceutical Chemistry, Faculty of Pharmacy, Badr University in Cairo (BUC), Cairo, Egypt

**Keywords:** natural molecules, hop cones, natural anxiolytics, DFT, molecular dynamics, RMSF analysis, molecular docking

## Abstract

This research work aimed to identify the main components that are responsible for the sedative properties of hop cones and allocate their targets. This investigation was performed through molecular docking, molecular dynamic simulations, root mean square fluctuation (RMSF) analysis, and DFT calculation techniques. The tested compounds from *Humulus lupulus* were compared to diazepam and paroxetine. Molecular docking showed that two-thirds of the compounds had a good affinity to gamma-aminobutyric acid (GABA), outperforming diazepam, while only three surpassed paroxetine on the SERT. Compounds 3,5-dihydroxy-4,6,6-tris(3-methylbut-2-en-1-yl)-2-(3-methylbutanoyl)cyclohexa-2,4-dien-1-one (**5**) and (*S,E*)-8-(3,7-dimethylocta-2,6-dien-1-yl)-5,7-dihydroxy-2-(4-hydroxyphenyl)chromen-4-one (**15**) showed stable binding and favorable energy parameters, indicating their potential for targeting GABA receptors and the SERT. This study provides a basis for future clinical research on these promising compounds.

## 1 Introduction

Anxiety is pathologic when it becomes maladaptive, permanent, and unmanageable and interferes with daily life. The current standard of care for anxiety disorders is based mostly on psychotherapy. Medications for anxiety disorders are now available, but they fall short of ideal in terms of efficacy and acceptability. Noncompliance, inadequate response to treatment, and relapse are serious problems for individuals who obtain treatment. Collectively, new effective methods for treating anxiety and associated diseases are desperately needed (Sartori and Singewald, 2019).

Serotonin (5-HT), a well-known neurotransmitter involved in regulating emotions, plays a crucial function in the neurobiology of anxiety (Zangrossi et al., 2020). The serotonin transporter (SERT) ends serotonergic signaling by actively transporting the neurotransmitter back into the presynaptic neurons in a sodium- and chloride-dependent fashion (Holmes et al., 2003). The SERT serves as a target for antidepressant and psychostimulant medications, which inhibit reuptake and extend the duration of neurotransmitter signaling. Selective serotonin reuptake inhibitors such as paroxetine, escitalopram, sertraline, and fluoxetine, are currently first-line treatment medications for most anxiety disorders, with a superior benefit/risk ratio than any other form of the available pharmacotherapy (Sartori and Singewald, 2019; Gosmann et al., 2021).

Similarly, gamma-aminobutyric acid (GABA), the principal inhibitory neurotransmitter in the central nervous system, assumes paramount importance in anxiety regulation (Kalueff and Nutt, 2007; Gauthier and Nuss, 2015). GABAergic neurotransmission, chiefly mediated through GABA-A receptor subtypes, orchestrates anxiolytic effects via dampening excitatory neuronal activity (Möhler, 2012). Pharmacological agents such as benzodiazepines (diazepam) potentiate GABA receptor function, eliciting sedative and anxiolytic responses (Inada et al., 2003). Nonetheless, protracted usage of benzodiazepines entails risks of tolerance and dependence, prompting exploration of alternative medications like gabapentinoids, which also modulate the GABA system. Therapeutic interventions targeting GABA receptors aspire to restore neural equilibrium, alleviate anxiety symptomatology, and ameliorate overall mental wellbeing (Hollister, 1981; Michelini et al., 2007).

**FIGURE 1:**
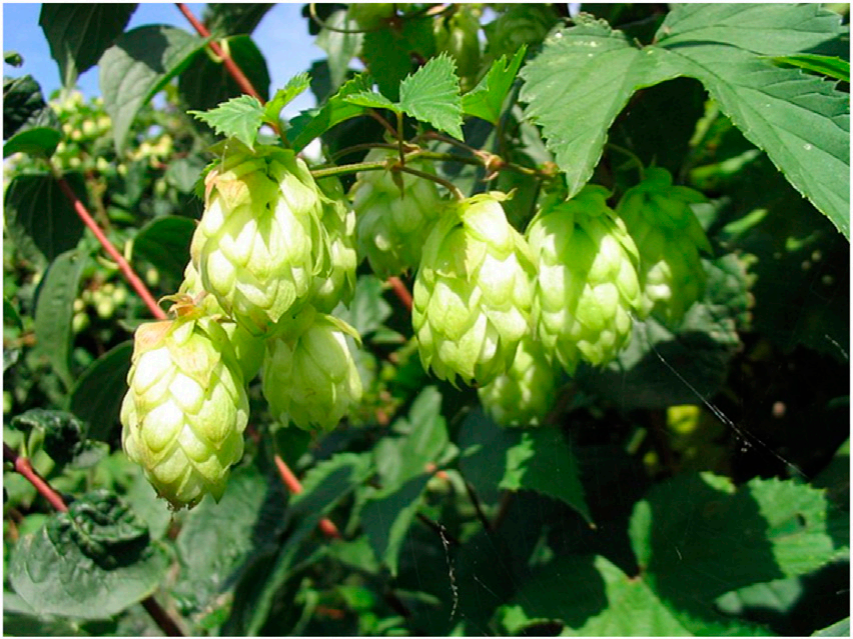
Used parts of *Humulus lupulus L.* retrieved from https://en.wikipedia.org/wiki/Humulus_lupulus.

Plants and their derivatives make up a significant portion of the human diet. Exploring the healing effects of plants should thus remain the primary focus of ongoing research due to their little or no negative side effects. Furthermore, synergistic effects can significantly improve their action or the action of current medications and therapies (Knez Hrnčič et al., 2019). *Humulus lupulus L*. (common hop) is a perennial herbaceous liana and one of three *Humulus* species in the *Cannabaceae* family. Strobili (hops) are cone-like structures in female plants (Korpelainen and Pietiläinen, 2021). The hop plant, seen in Figure 1, has been in continuous use for years, if not millennia, primarily as an antimicrobial component in beer. It is now used to treat agitation, anxiety, and sleep issues (Heinlein et al., 2014; Hrnčič et al., 2019). The first isolated phytoconstituent was lupulin, and it was considered almost a specific remedy for scrofula, struma, and various skin diseases (REILLY, 1906). In addition to lupulin, hop contains a variety of phytochemicals including xanthohumol, humulone, lupulone, 8-prenylnaringenin, and myrcene, which contribute to its antioxidant and antimicrobial properties. Due to the abundance of dietary phytochemicals in *H. lupulus* that have medical uses, including antibacterial, antioxidant, anticancer, antiplatelet, antidiuretic, anti-inflammatory, and sedative effects, this plant has received special attention (Tronina et al., 2020). To confirm these physiological effects, several *in vitro* and *in vivo* investigations have been carried out (Heinlein et al., 2014). Research driven by these effects revealed that these neurological effects may be partially mediated through the modulation of GABA receptors (Benkherouf et al., 2020; Carbone and Gervasi, 2022).

**FIGURE 2:**
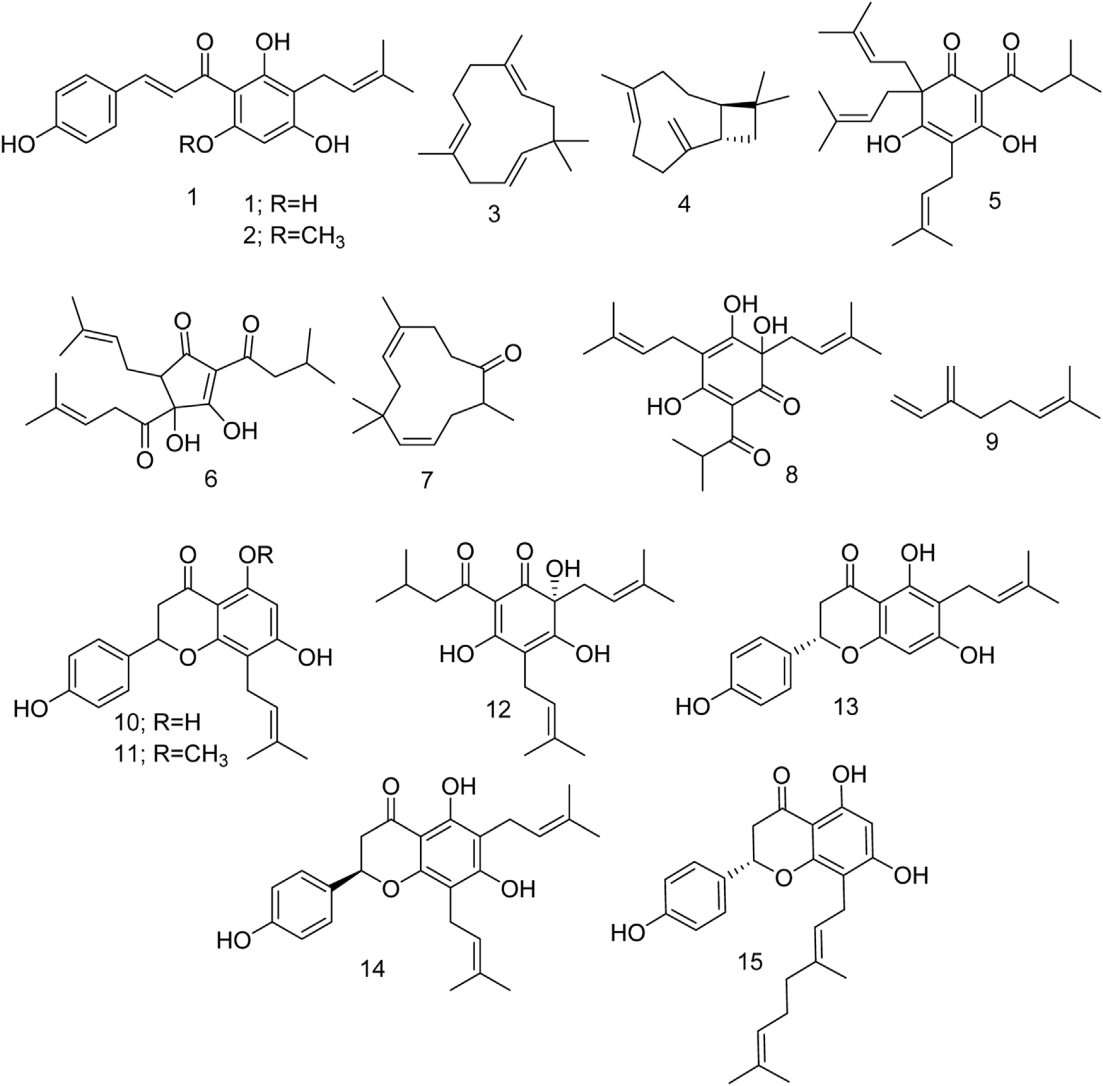
Different compounds isolated from *Humulus lupulus L*.

Encouraged by these data, we explored the potential of various phytochemicals (Figure 2) isolated from *H. lupulus L*. As such, after a literature search, we compiled 15 compounds and studied their effects on GABA and the human serotonin transporter (SERT) through multiple *in silico* techniques.

## 2 Materials and methods

### 2.1 Molecular docking

GABA and SERT receptors were downloaded from the Protein Data Bank (PDB IDs: 6X3X and 5I6X, respectively) (Coleman et al., 2016; Kim et al., 2020). A literature search was performed to identify isolated compounds from *Humulus lupulus L.*, and PubChem (https://pubchem.ncbi.nlm.nih.gov/) was accessed to obtain the smiles (Kim et al., 2021). The compounds were compiled into a database for processing, in addition to the receptors, and all were prepared and optimized using the standard protocol of the Autodock Vina. Energy minimization was performed using M.G.L. tools, and the active site was set to the pocket surrounding the co-crystalized ligands (diazepam and paroxetine) (Trott and Olson, 2010). Docking was performed using the Autodock vina with its scoring function. The docking site was selected as 24*24*24 Å surrounding co-crystallized ligands. Validation was achieved by re-docking of the co-crystallized ligands into their binding pocket, followed by calculating the RMSD between poses. Biovia DS Visualizer was used to analyze the docking results as well (Dassault Systèmes, 2021; Belal et al., 2022a).

### 2.2 Dynamic simulations and calculations

The Schrödinger Desmond package was used for molecular dynamics simulations of free proteins and their complexes (Bowers et al., 2006). Preparation was done using the “OPLS4” force field as described before. The system was constructed using “TIP3P” water molecules in an orthorhombic box (Lu et al., 2021). The systems underwent simulation under the default settings of “NPT” ensemble (300 K and 1.01325 bar) for 50 ns for each simulation, followed by an interaction analysis to calculate the RMSD, RMSF, and other properties (Schrödinger, 2021; Belal et al., 2022b).

### 2.3 Molecular mechanics-generalized Born surface area calculations

The MM-GBSA technique was used to compute the binding free energy of the studied protein–ligand complexes, which integrated molecular mechanics (MM) force fields with a generalized Born and surface area continuum solvation model using the Schrodinger Prime package (Jacobson et al., 2004; Belal et al., 2022c). Contributions from molecular mechanics energies and polar and non-polar solvation were estimated using the equation ΔE_B_ = ΔE_C_ – (ΔE_P_ +ΔE_L_), where ΔE_B_ is the calculated binding free energy of the complex, ΔE_C_ is the binding free energy of the complex, ΔE_P_ is the binding free energy of the protein, and ΔE_L_ is the binding free energy of the ligand (Dimić et al., 2019; Jupudi et al., 2022; Jevtovic et al., 2023).

### 2.4 Density functional theory calculations

The Spartan '14 program was used to perform quantum chemistry calculations using the DFT method. Spartan '14 was used to display all of the data files. The density functional theory (DFT) at 6-311G++(d,p) basis set/B3LYP approach was utilized to optimize the organic chemical structure of the compound under investigation, and Chem3D 15.0 software was used to create the original chemical structure (Legler et al., 2015).

## 3 Results and discussion

### 3.1 Molecular docking

**FIGURE 3:**
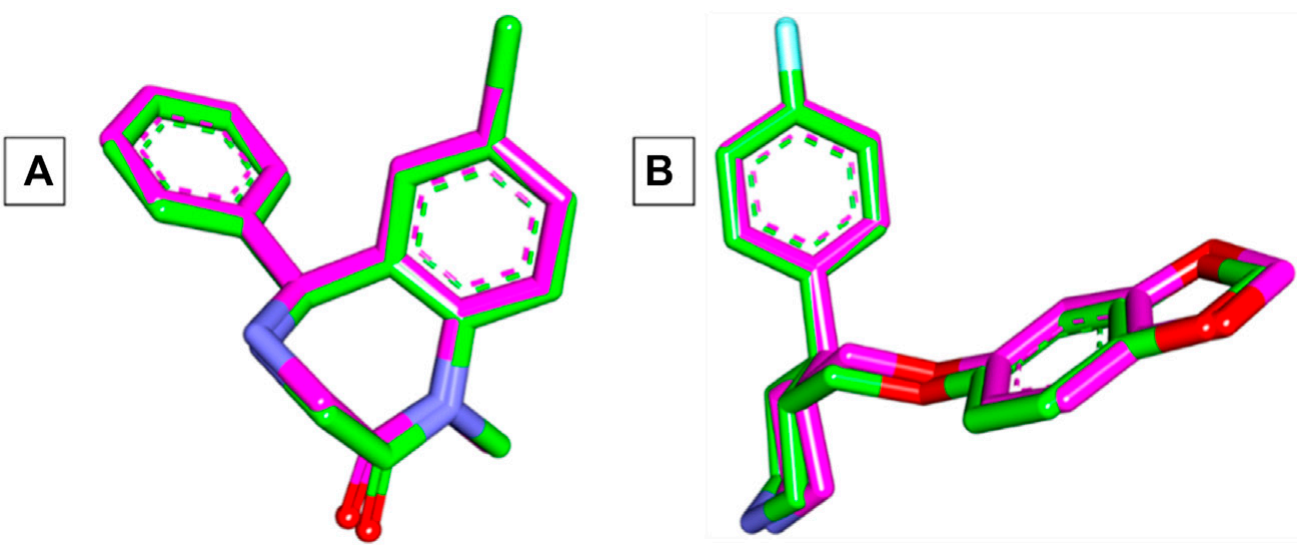
**(A)** Superimposition of co-crystallized (green) and docked (pink) diazepam in the GABA receptor showing an RMSD value of 0.17 Å. **(B)** Superimposition of co-crystallized (green) and docked (pink) paroxetine in the SERT receptor showing an RMSD value of 0.33 Å.

The Autodock Vina program was used in the current docking study. Validation of docking accurately reproduced the binding conformation of the co-crystallized ligands with GABA and human serotonin transporter receptors (PDB IDs: 3X6X and 5I6X, respectively). In both cases, the monomer of the receptor was obtained and processed for subsequent analysis. The RMSD values were calculated between the co-crystallized poses and the docked poses of the same ligands, and the results revealed minor deviations of 0.17 and 0.33 Å for GABA and 5-HT, respectively Figure 3. These results indicated the validity of the docking studies, and the compounds were docked accordingly. As shown in Table 1, the compounds exhibited negative binding scores, indicating the favorability of their binding to both GABA and SERT receptors. Additionally, for comparative analysis, both diazepam and paroxetine were used as references for both targets.

**TABLE 1:** Docking results of hop cones compounds against GABA and SERT receptors in kcal/mole.

No.	Compound chemical name	CID	GABA	SERT
**1**	(*E*)-3-(4-hydroxyphenyl)-1-(2,4,6-trihydroxy-3-(3-methylbut-2-en-1-yl)phenyl)prop-2-en-1-one	6443339	−7.53	−7.42
**2**	(*E*)-1-(2,4-dihydroxy-6-methoxy-3-(3-methylbut-2-en-1-yl)phenyl)-3-(4-hydroxyphenyl)prop-2-en-1-one	639665	−8.1	−8.15
**3**	(1*E*,4*E*,8*E*)-2,6,6,9-tetramethylcycloundeca-1,4,8-triene	5281520	−6.04	−5.95
**4**	(1*R*,9*S*,*E*)-4,11,11-trimethyl-8-methylenebicyclo [7.2.0]undec-4-ene	5281515	−6.16	−5.92
**5**	3,5-dihydroxy-4,6,6-tris(3-methylbut-2-en-1-yl)-2-(3-methylbutanoyl)cyclohexa-2,4-dien-1-one	68051	−9.23	−9.05
**6**	3,4-dihydroxy-5-(3-methylbut-2-en-1-yl)-2-(3-methylbutanoyl)-4-(4-methylpent-3-enoyl)cyclopent-2-en-1-one	93090	−8.39	−8.02
**7**	(4*Z*,8*Z*)-2,6,6,9-tetramethylcycloundeca-4,8-dien-1-one	101297706	−5.84	−6.33
**8**	3,5,6-trihydroxy-2-isobutyryl-4,6-bis(3-methylbut-2-en-1-yl)cyclohexa-2,4-dien-1-one	196915	−8.19	−8.02
**9**	7-methyl-3-methyleneocta-1,6-diene	31253	−5.42	−5.49
**10**	5,7-dihydroxy-2-(4-hydroxyphenyl)-8-(3-methylbut-2-en-1-yl)chroman-4-one	513197	−8.62	−8.52
**11**	7-hydroxy-2-(4-hydroxyphenyl)-5-methoxy-8-(3-methylbut-2-en-1-yl)chroman-4-one	480764	−8.29	−8.14
**12**	(*R*)-3,5,6-trihydroxy-4,6-bis(3-methylbut-2-en-1-yl)-2-(3-methylbutanoyl)cyclohexa-2,4-dien-1-one	442911	−8.33	−7.99
**13**	(*S*)-5,7-dihydroxy-2-(4-hydroxyphenyl)-6-(3-methylbut-2-en-1-yl)chroman-4-one	155094	−8.04	−7.76
**14**	(*R*)-5,7-dihydroxy-2-(4-hydroxyphenyl)-6,8-bis(3-methylbut-2-en-1-yl)chroman-4-one	124035	−8.90	−8.74
**15**	(*S,E*)-8-(3,7-dimethylocta-2,6-dien-1-yl)-5,7-dihydroxy-2-(4-hydroxyphenyl)chroman-4-one	6475921	−8.76	−9.12
Diazepam		3016	−6.83	---
Paroxetine		43815	---	−8.71
CID: PubChem identification code				

### 3.1.1 Molecular docking against GABA receptors

**FIGURE 4:**
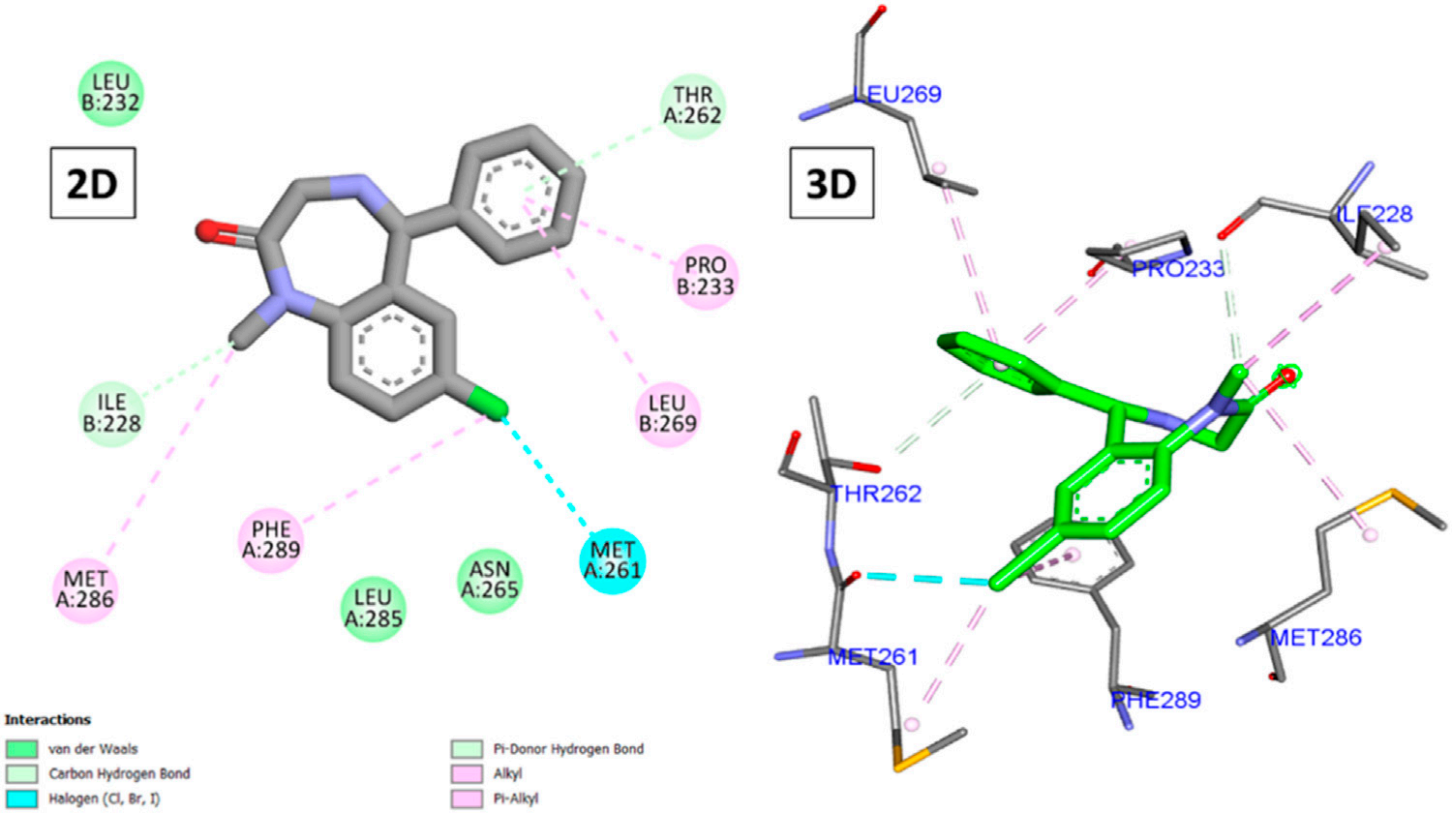
2D and 3D interactions of diazepam with GABA.

Analysis of the binding of diazepam (Figure 4) revealed its low score (−6.83 kcal/mole) due to the formation of several hydrophobic interactions through its chlorophenyl and phenyl moieties with Ile228, Pro233, Met261, Leu269, Met286, and Phe289, in addition to two hydrogen bonds with Ile228 and Thr262. Most of the compounds (two-thirds) achieved higher scores than diazepam, with the top-scoring compounds being **5**, **14**, **15**, and **10** (−9.23, −8.90, −8.76, and −8.62 kcal/mole, respectively).

**FIGURE 5:**
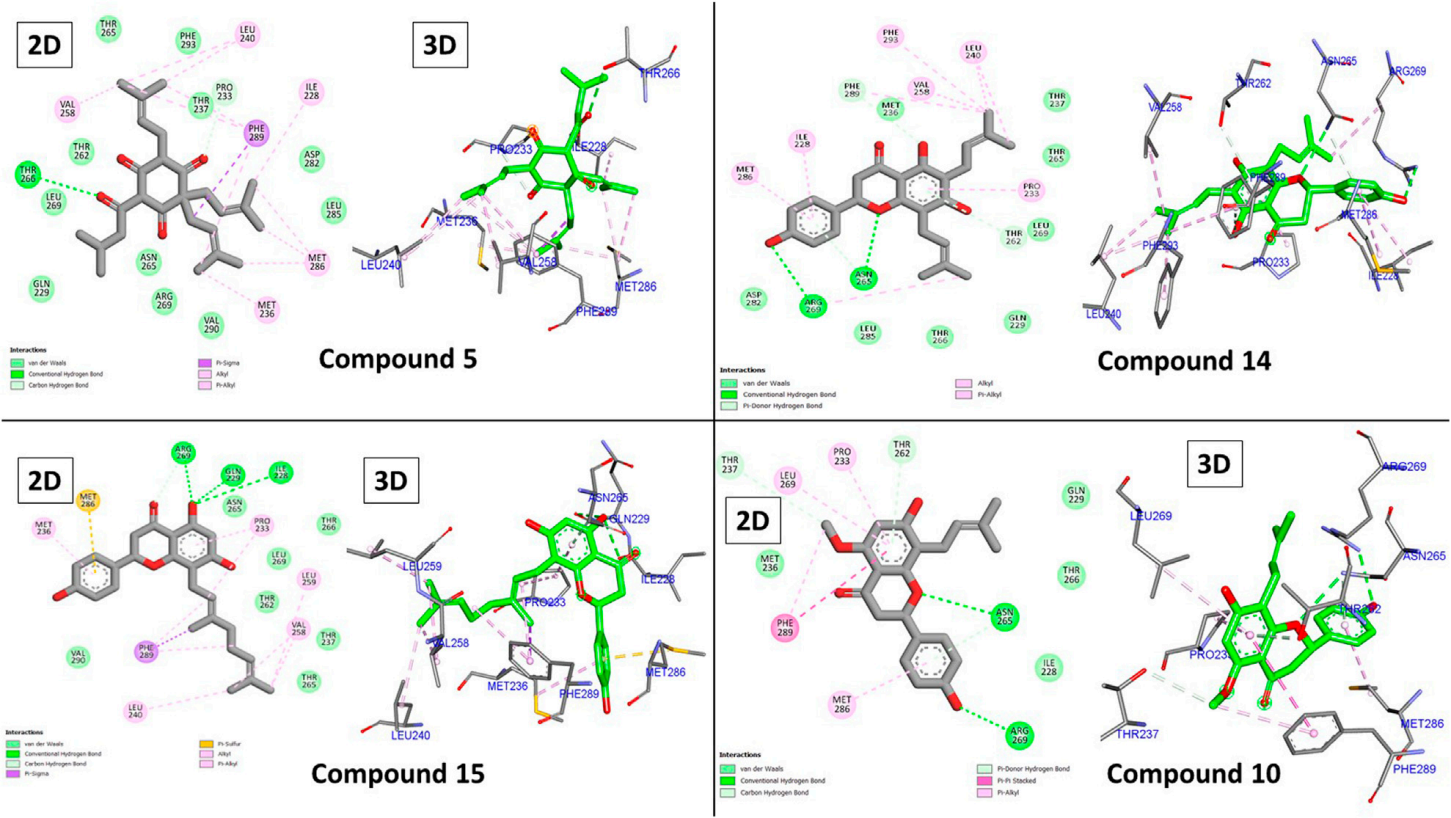
2D and 3D binding interactions of compounds **5**, **14**, **15**, and **10**.

The hydrophobic groups in our top three compounds played an important role in their binding, as shown in Figure 5. Similar to diazepam, all top three compounds formed many hydrophobic interactions with one or two hydrogen bonds. Compound **5**’s superior binding is attributed to its three 3-methylbut-3-enyl moieties that exclusively interacted with Ile228, Met236, Leu240, Val258, Met286, Phe289, and Phe293 through twelve hydrophobic bonds. Additionally, two hydrogen bonds were observed as well with Pro233 and Thr266 through two carbonyls.

**FIGURE 6:**
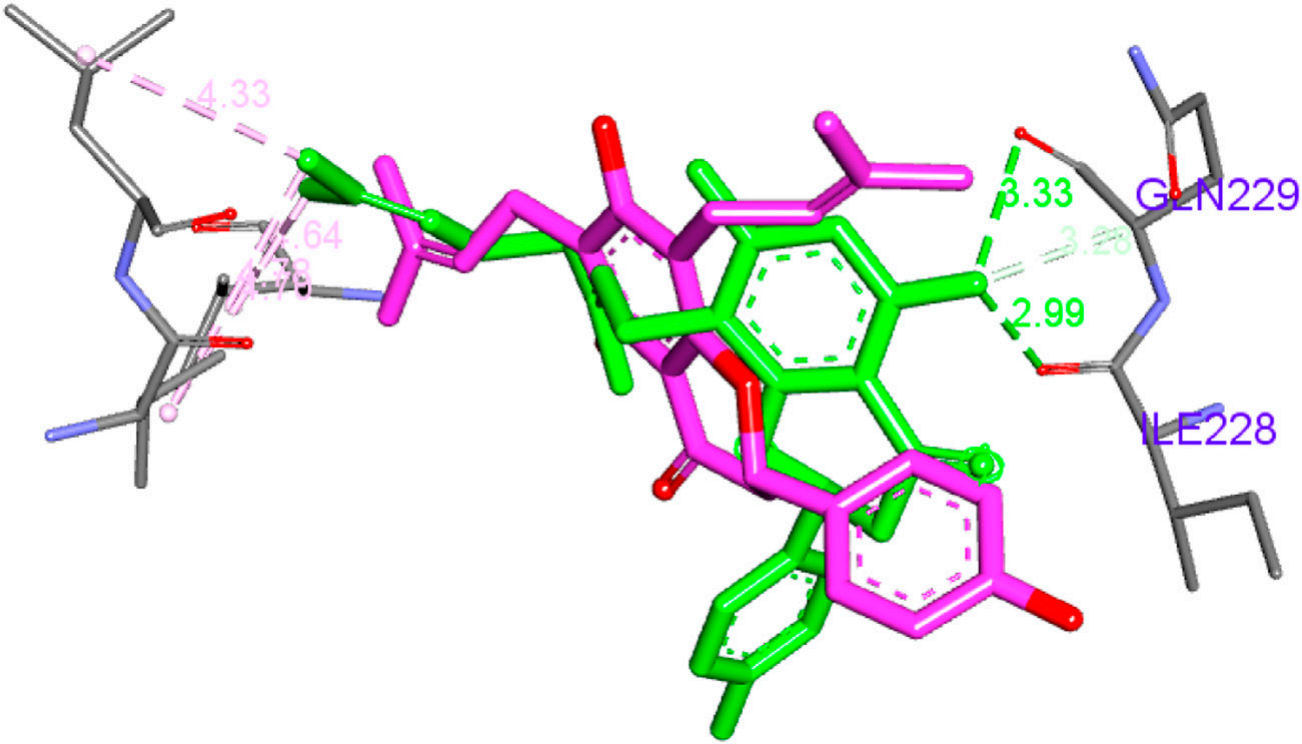
Superposition of compounds **14** and **15**.

Similarly, the same amino acids formed hydrophobic bonds with compound **14** and one hydrogen bond with Arg269. Unlike compound **5**, compound **14** possessed only two 3-methylbut-3-enyl moieties, which contributed with only six hydrophobic interactions. However, it was compensated through its central chromone and terminal phenyl rings with additional three bonds. Compound **15** behaved similarly to **14**; its central chromone and terminal phenyl rings oriented toward the same amino acids. The main difference is the hydrophobic counterpart, the 3,7-dimethyloctane-2,6-dienyl group. This group’s length forces the orientation of the chromone ring to flip to accommodate the binding pocket. This in turn brings the central chromone ring in close proximity to Ile228 and GLN229 to form hydrogen bonds through the hydroxyl group Figure 6. Finally, compound **10** behaved similarly to **14** but with only one main hydrophobic moiety (3-methylbut-3-enyl), which explains its inferior score compared to that of the others.

### 3.1.2 Molecular docking against SERT receptors

**FIGURE 7:**
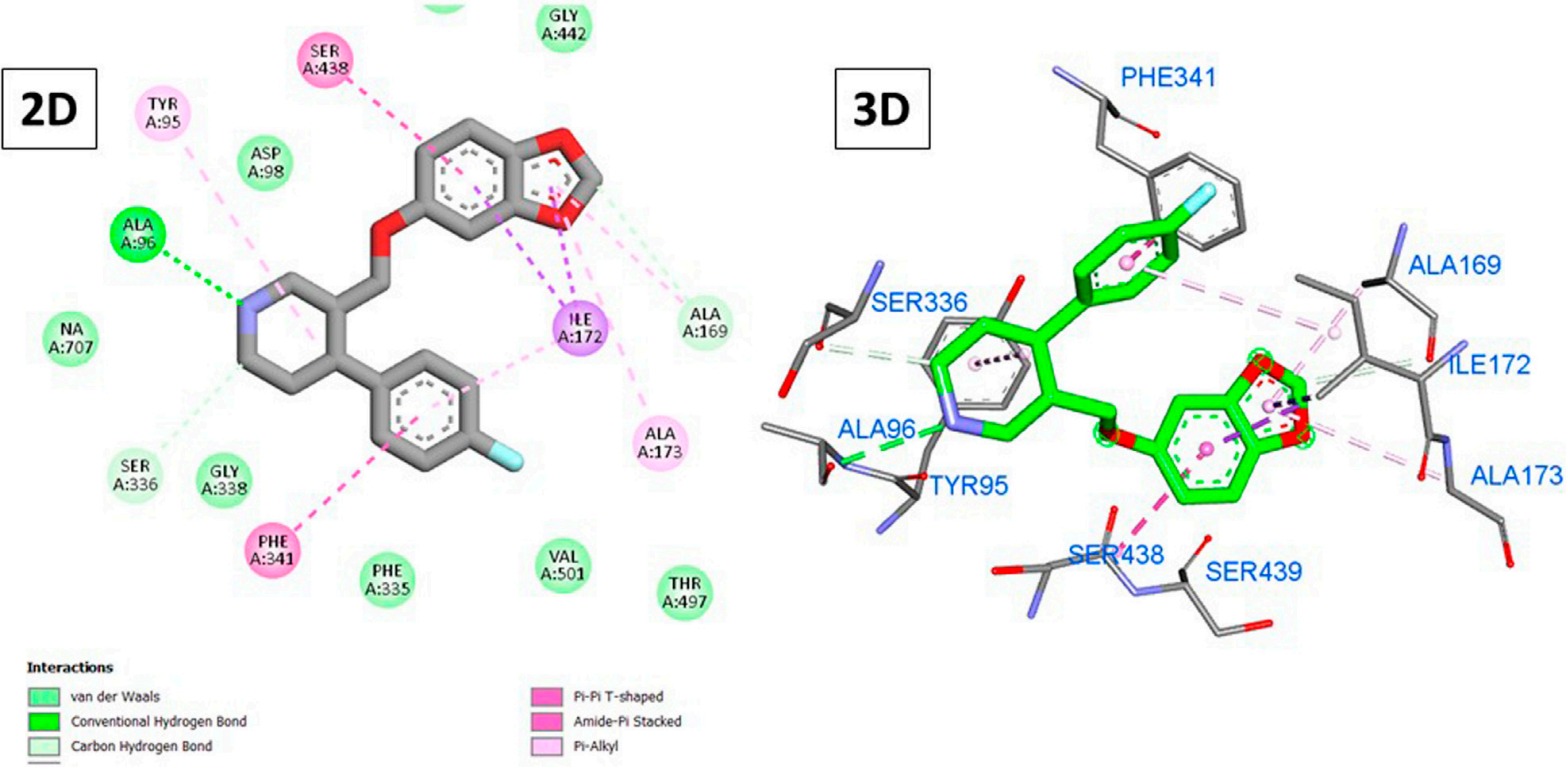
2D and 3D interactions of paroxetine with the SERT.

On the other hand, paroxetine (Figure 7) exhibited a score of −8.71 kcal/mole and interacted with Tyr95, Ala169, Ile172, Ala173, Phe341, and Ser438 through hydrophobic interactions with its fluorophenyl and 1,3-benzodixole moieties in addition to three hydrogen bonds with Ala69, Ala169, and Ser336 mainly through the piperidine ring. Unlike previously, only three compounds exhibited higher scores than paroxetine, with the top-scoring compounds being **15**, **5**, and **14** (−9.12, −9.05, and −8.74 kcal/mole, respectively).

**FIGURE 8:**
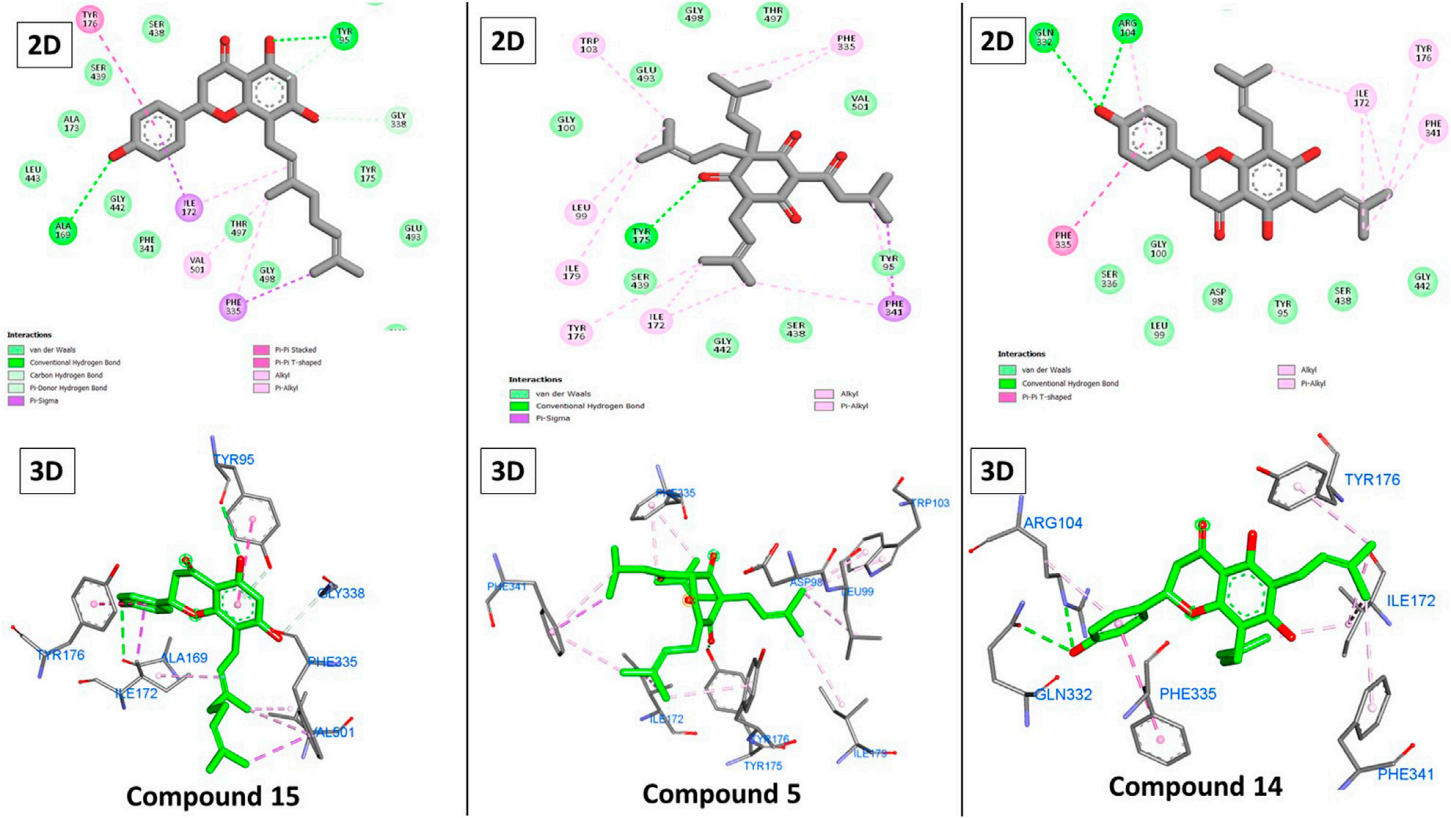
2D and 3D binding of **15**, **5**, and **14** to the SERT.

Again, the presence of hydrophobic alkenyl chains like 3-methylbut-3-enyl and 3,7-dimethyloctane-2,6-dienyl groups impacted the binding of the compounds. These chains formed several hydrophobic interactions with Ile172, Tyr176, and Phe335 (Figure 8). Overall, the three compounds possessed these chains, but the individual differences can be attributed to the difference in hydrogen bonding. Individually, compound **15**’s strongest binding was attributed to the three additional hydrogen bonds with Tyr95, Ala169, and Gly338 through its three hydroxy groups on the coumarin and terminal phenyl groups. Although compound **5** formed only one hydrogen bond with Tyr175, its ability to bond via more hydrophobic interactions with other amino acids (Leu99, Trp103, Ile179, and Phe341) compensated for this shortcoming and explained the moderate decrease in its score. Alternatively, the lack of some hydrophobic interactions explained how compound **14** achieved poorer results, despite two hydrogen bonds with Arg104 and Gln332.

The interesting results of compounds **5** and **15** over both targets established their potential for further analysis throughout our study.

### 3.2 Dynamic simulations and calculations

Molecular dynamic simulations extensively analyzed the binding modes and stability under realistic physiological conditions. Using the Schrodinger Maestro suite, the proteins with and without compounds **5** and **15** were simulated for 50 ns. Additionally, diazepam and paroxetine were simulated with their respective proteins for comparison. Afterward, the simulation trajectories were analyzed, and several attributes were calculated, such as the root mean square deviation (RMSD) of the protein–ligand complex for determination of the binding interaction stability, the RMSD of ligands to evaluate the conformational changes ligands undergo over the estimated simulation process, as well as the root mean square fluctuation (RMSF) of the amino acid residues and their contact with ligands.

#### 3.2.1 RMSD analysis

**FIGURE 9:**
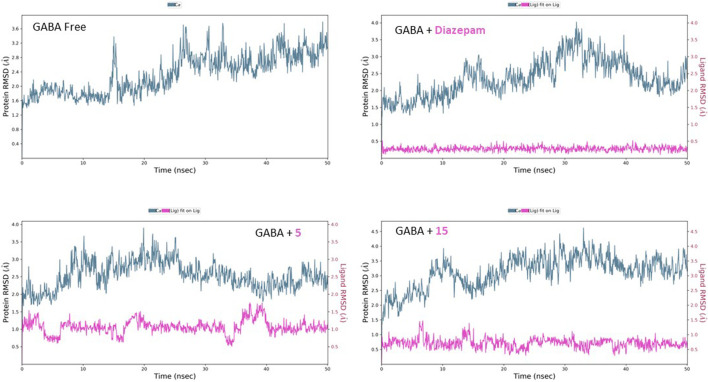
RMSD analysis of free GABA and GABA in complex with diazepam, compound **5** *,* and compound **15** throughout the 50 ns simulation time.

The free GABA protein shows relative homogeneity in behavior, as demonstrated by the RMSD’s initial consistency around 2 Å for the first 25 ns, followed by a plateau at 2.80 Å (Figure 9). After introducing diazepam, the RMSD behavior was lowered slightly to approximately 2.50 Å. The effect of binding of both compounds **5** and **15** on the protein can be seen similarly, with the RMSD fluctuating at approximately 2.40 and 2.90 Å, respectively. Moreover, the inspection of both ligands’ conformational motion showed that compounds **5** and **15** maintain high conservation and stability, evidenced by the uniform RMSD at 1.00 and 0.50 Å, respectively.

**FIGURE 10:**
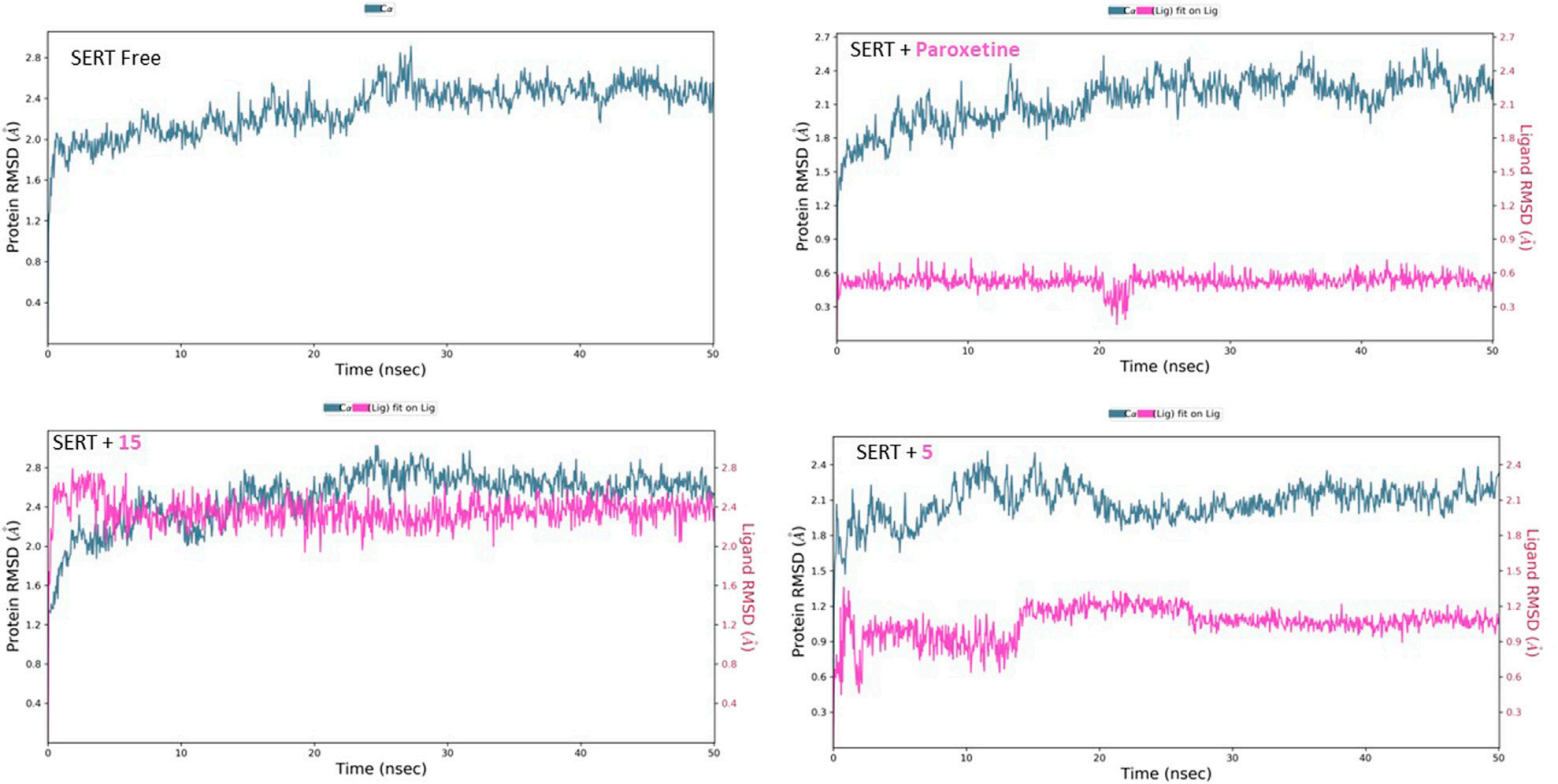
RMSD analysis of free SERT and SERT in complex with paroxetine, compound **5**, and compound **15** throughout the 50 ns simulation time.

Similarly, the free SERT showed relative homogeneity in behavior, as demonstrated by the RMSD’s initial consistency around 2.10 Å, followed by a plateau at 2.50 Å (Figure 10). The binding of paroxetine altered the RMSD behavior, lowering it slightly to approximately 2.40 Å. The binding of compound **15** affected the protein’s RMSD, similar to that of paroxetine lowering, while compound **5** decreased it slightly to approximately 2.10 Å. Moreover, the inspection of both ligands’ conformational motion showed that compounds **15** and **5** maintain relative conformational stability with a uniform RMSD at 2.40 and 1.10 Å, respectively.

#### 3.2.2 RMSF analysis

**FIGURE 11:**
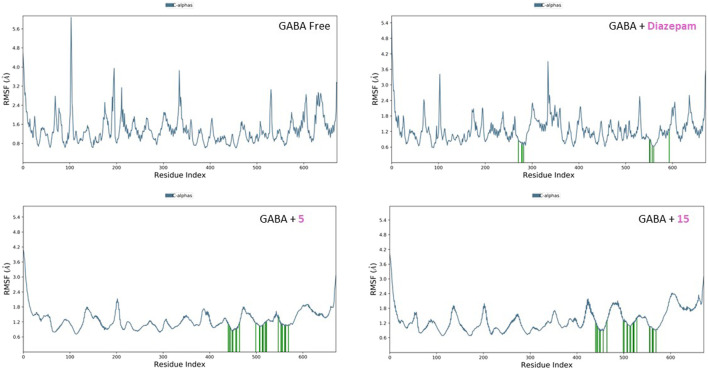
RMSF analysis of free GABA and GABA in complex with diazepam, compound **5**, and compound **15** throughout the 50 ns simulation time.

**FIGURE 12:**
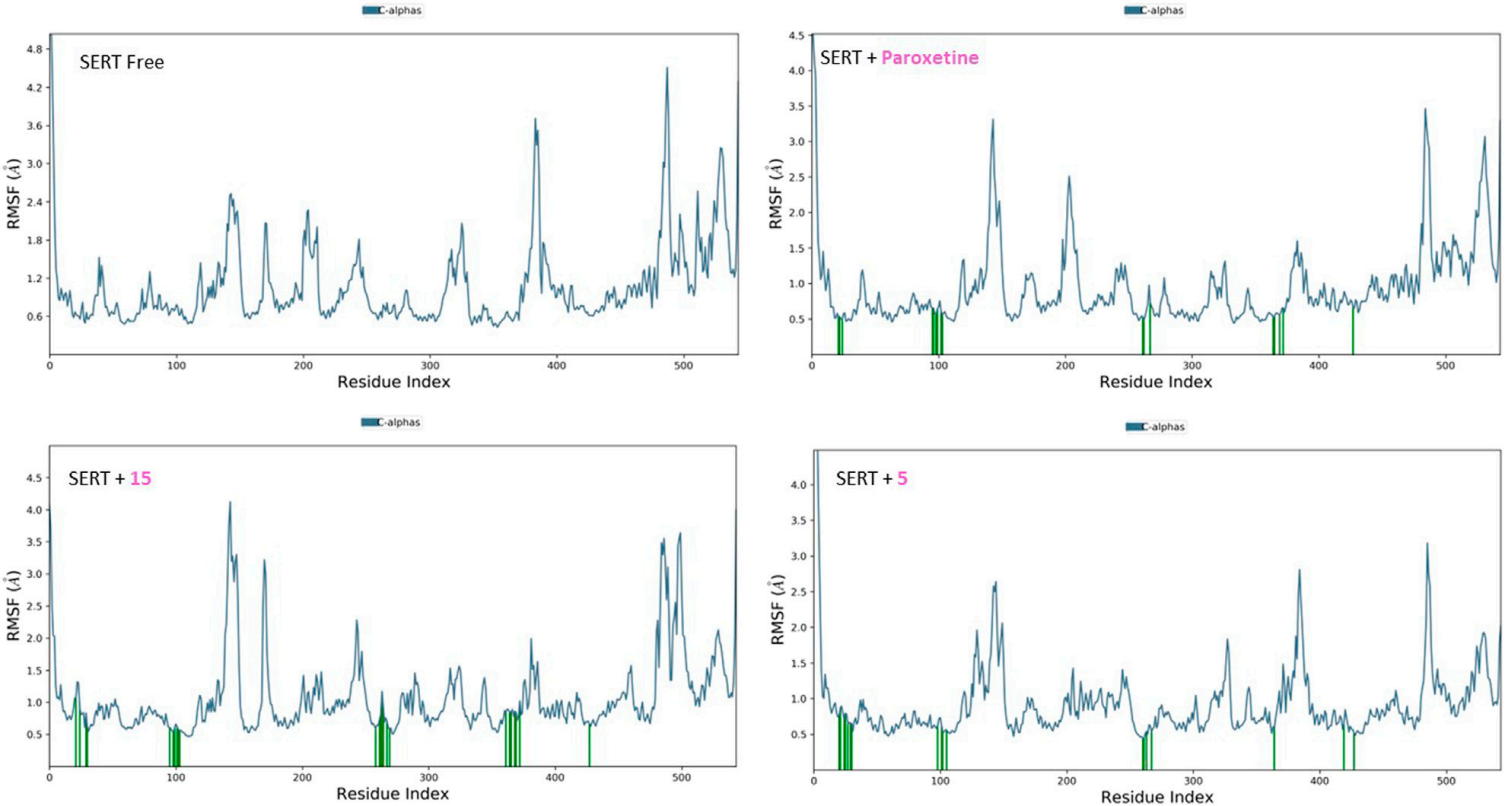
RMSF analysis of free SERT and SERT in complex with paroxetine, compound **5**, and compound **15** throughout the 50 ns simulation time.

The RMSF plots for the free proteins and their complexes with both compounds were assessed to expand our understanding of their interactions throughout the 50-ns simulation (Figures 11, 12). The RMSF measures the flexibility of each residue in the protein, providing insights into how ligand binding affects protein dynamics.

For the free GABA protein, the RMSF plots indicated a high degree of flexibility, suggesting that the protein exhibits significant conformational freedom in the absence of a ligand. However, upon binding with diazepam, compounds **5** and **15** showed a marked reduction in the fluctuations of several residues. This indicates that these ligands stabilize the protein structure by restraining its dynamic behavior. Similarly, the RMSF analysis of the SERT revealed high residue flexibility in its unbound state. The introduction of paroxetine, compound **5**, and compound **15** led to decreased fluctuations in SERT residues, suggesting that these ligands also stabilize the transporter structure, reducing its conformational flexibility.

These observations align with previous molecular docking results, which suggested strong binding interactions between ligands and proteins. The reduction in residue fluctuations upon ligand binding supports the stability of the interactions.

#### 3.2.3 MM-GBSA calculations

Molecular mechanics-generalized Born surface area is one of the most frequent methods for determining the binding free energy (MM-GBSA). This approach integrates molecular mechanics (MM) force fields with the generalized Born (GB) and surface area (SA) continuum solvation model to provide an estimation of the binding free energies, offering insights into the stability and affinity of the complexes. The calculations were conducted using the Schrödinger Prime package, a comprehensive tool for performing these sophisticated computations. The lower the predicted binding free energy of a ligand–protein complex, the more stable the complex will be, and the greater the ligand’s activity and potency. Both complexes showed stable binding throughout the dynamic simulation, as demonstrated by the energy scores in Table 2.

**TABLE 2:** MM-GBSA results of compounds **5** and **15** when complexed with GABA and SERT receptors.

	GABA				SERT			
	5		15		5		15	
	Start	End	Start	End	Start	End	Start	End
**ΔG**_Binding_	−86.79	−70.87	−72.12	−74.69	−63.85	−69.10	−73.16	−69.66
**ΔG**_Binding_ _Coulomb_	−12.06	−3.94	−12.87	−12.04	−8.27	−4.02	−13.48	−14.75
**ΔG**_Binding (NS)_	−92.82	−76.56	−82.54	−77.26	−68.99	−74.77	−80.68	−74.33
**ΔG**_Binding (NS)_ _Coulomb_	−13.40	−3.97	−13.77	−11.00	−7.46	−4.15	−13.58	−16.76

### 3.3 DFT calculations

**FIGURE 13:**
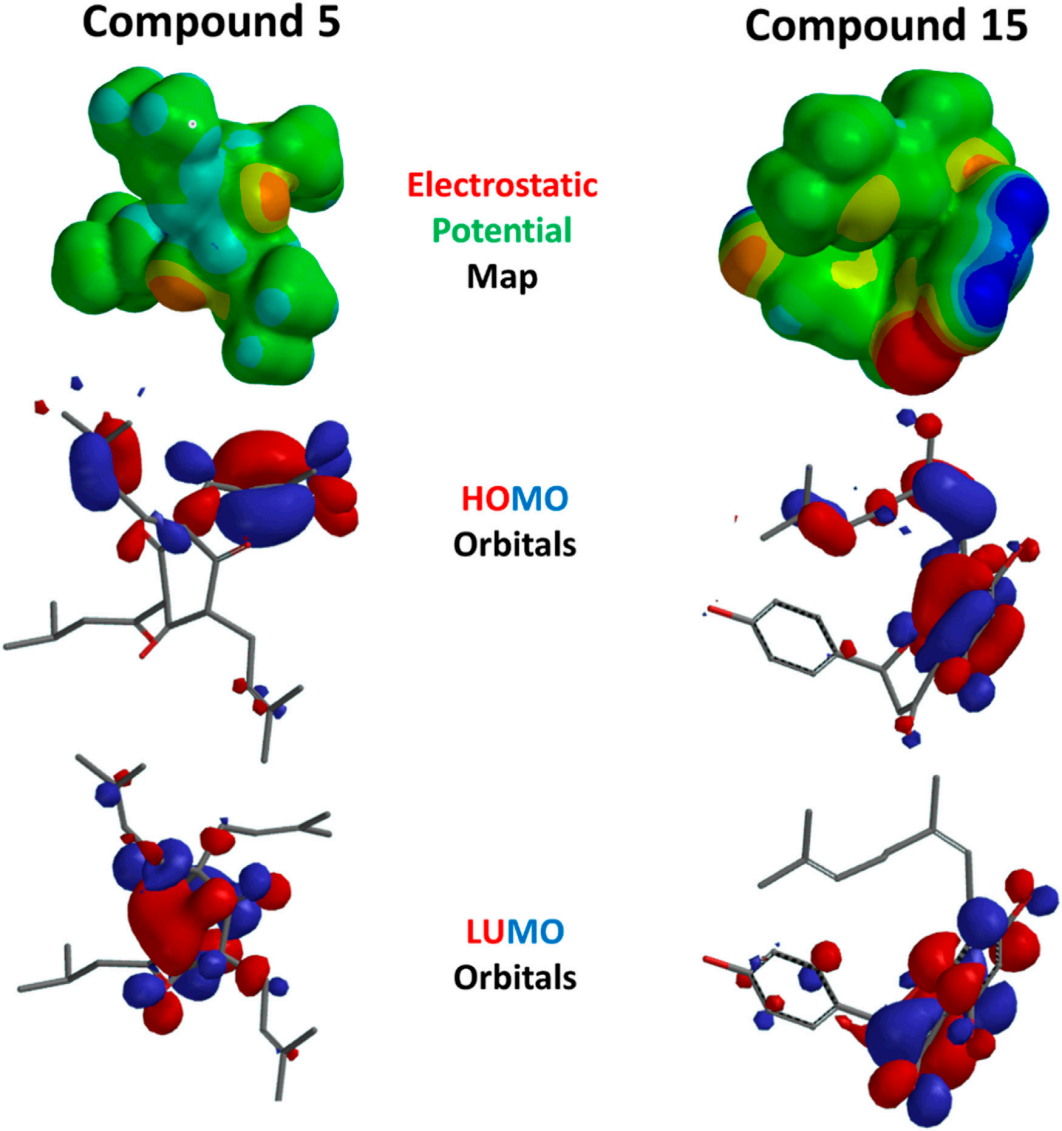
Electrostatic potential map and HOMO and LUMO orbitals of compounds **5** and **15**.

The DFT/B3LYP approach was used in the current work to perform quantum chemical computations to optimize the selected structures. The DFT (B3LYP) method with the 6-311G++(d,p) basis set was applied in this test. The optimized structure and its HOMO and LUMO values are represented in Figure 13. The HOMO energy expresses the ability of the compound to give electrons as an electron donor. It is localized mainly on the two 3-methylbut-2-en-1-yl moieties of compound **5** on carbon 6. This electronic enrichment explains their hydrophobic capacity to form multiple interactions within SERT and GABA receptors, as previously shown in docking. It was mostly localized on the dihydroxyphenyl ring with its dangling eight-carbon substituent in compound **15**. This also impacted the hydrophobic binding of the alkenyl tail and allowed for better availability of the hydroxyl group to form hydrogen bonds.

On the other hand, the LUMO energy displayed by a site can act as an electron attractor, i.e., electron acceptors, due to vacant orbitals and localized at the central six-membered ring(s) of compounds **5** and **15**. Similarly, the electrostatic potential maps of the compounds showed areas with electron localization throughout the molecules, with red and blue representing electron-rich (negative) and -deficient (positive) localizations, respectively. Finally, the DFT calculations revealed favorable energetic parameters for the selected compounds Table 3.

**TABLE 3:** DFT calculation results of compounds **5** and **15**.

	Energy (au)	Energy solvation (kj/Mol)	E Homo (ev)	E Lumo (ev)	Dipole moment (debye)	No. of conformers
**5**	−1,314.87133	−5.58	−6.59	−1.82	3.65	157,464
**15**	−1,345.97759	−63.96	−6.06	−1.45	3.54	5,184

## 4 Conclusion

The sedative properties of hop cones (*H. lupulus L*.) have been extensively exploited in herbal remedies for a long time. Despite this, there is no research to isolate the main component responsible for these properties and allocate its target. Thus, we analyzed the isolated compounds from *H. lupulus L*. and studied their effects compared to those of two widely used drugs, diazepam and paroxetine, using multiple *in silico* techniques. Initially, molecular docking against GABA and SERT receptors was performed to identify their potential against both targets. As a result, nearly two-thirds of the compounds demonstrated good affinity to GABA, even outperforming diazepam. Alternatively, only three compounds could reach scores more than that of paroxetine itself on the SERT. In both targets, compounds **5** and **15** were among the top-performing compounds with docking scores equally (−9.23 and −8.76 Kcal/mole for GABA and −9.05 and −9.12 Kcal/mole for the SERT, respectively) surpassing those of the two drugs used as references (diazepam and paroxetine). They showed scores of −6.83 and −8.71 kcal/mole for GABA and the SERT, respectively and were promoted for further analysis. Subsequent analysis was done to evaluate their binding under normal physiological conditions for an extended time, reaching 50 ns. Both compounds demonstrated uniform and stable binding throughout the simulation time, compared to diazepam and paroxetine with RMSD values of approximately 2.40–2.90 Å and 2.10–2.50 Å for GABA and the SERT, respectively. Additionally, the RMSF behavior observed across the different simulations was consistent with both drugs. Further supporting evidence is their MM-GBSA energy calculation results, which enforced their stability as well. Finally, a DFT analysis was also performed to assess their stability, and both showed favorable energy parameters. In conclusion, this study demonstrates the potential of compounds **5** (lupulone) and **15** (chromen-4-one derivative) in targeting GABA and SERT receptors, while providing evidence and a basis for future clinical research.

## Data Availability

All data are included in the manuscript.
